# Long noncoding RNA DUXAP8 contributes to the progression of hepatocellular carcinoma via regulating miR‐422a/PDK2 axis

**DOI:** 10.1002/cam4.2861

**Published:** 2020-02-05

**Authors:** Feifei Wei, Liang Yang, Dandan Jiang, Min Pan, Guiyan Tang, Mingyue Huang, Jing Zhang

**Affiliations:** ^1^ Department of Oncology Fifth Clinical Medical College Guilin Medical University Guilin People's Republic of China; ^2^ Department of Oncology Jining NO.1 People's Hospital; Affiliated Jining NO.1 People's Hospital Jining Medical University Jining People's Republic of China

**Keywords:** DUXAP8, HCC, miR‐422a, PDK2

## Abstract

**Background:**

Hepatocellular carcinoma (HCC) is one of the most deadly cancer worldwide. Multiple long noncoding RNAs (lncRNAs) are recently identified as crucial oncogenic factors or tumor suppressors. In this study, we explored the functon and mechanism of lncRNA double homeobox A pseudogene 8 (DUXAP8) in the progression of HCC.

**Methods:**

Expression levels of DUXAP8 in HCC tissue samples were measured using qRT‐PCR. The association between pathological indexes and the expression of DUXAP8 was also analyzed. Human HCC cell lines SMMC‐7721 and QSG‐7701 were used in in vitro studies. CCK‐8 assay was used to assess the effect of DUXAP8 on HCC cell line proliferation. Scratch healing assay and Transwell assay were conducted to detect the effect of DUXAP8 on migration and invasion. Furthermore, dual‐luciferase reporter assay was used to confirm targeting relationship between miR‐422a and DUXAP8. Additionally, Western blot was used to detect the regulatory function of DUXAP8 on pyruvate dehydrogenase kinase 2 (PDK2).

**Results:**

DUXAP8 expression HCC clinical samples was significantly increased and this was correlated with unfavorable pathological indexes. High expression of DUXAP8 was associated with shorter overall survival time of patients. Its overexpression remarkably facilitated the proliferation, metastasis, and epithelial‐mesenchymal transition of HCC cells. Accordingly, knockdown of it suppressed the malignant phenotypes of HCC cells. Overexpression of DUXAP8 significantly reduced the expression of miR‐422a by sponging it, but enhanced the expression of PDK2.

**Conclusions:**

DUXAP8 was a sponge of tumor suppressor miR‐422a in HCC, enhanced the expression of PDK2 indirectly, and functioned as an oncogenic lncRNA.

## Graphical abstract

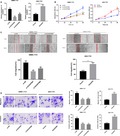

## 1 INTRODUCTION

Hepatocellular carcinoma (HCC) is one of the common tumors of the digestive system. Its high incidence and high mortality rate make it one of the major health threats in China.^1^, ^2^ Because HCC patients are asymptomatic in the early stage, most of them are diagnosed at an advanced stage with high mortality and low survival rate.^3^ At present, the treatment of HCC includes surgical treatment, chemotherapy, targeted drug therapy, etc, but due to frequent recurrence after surgery, chemoresistance and radioresistance, the prognosis of HCC patients is still not satisfactory.^4^, ^5^ Therefore, research on new biomarkers and new therapy targets for HCC is extremely important.

LncRNAs belong to noncoding RNAs that were once thought to be "noise" during transcription, but growing evidence suggests that they are not genomic "Garbage," but a functional RNA molecule that regulates the expression of genes in epigenetic regulation, transcriptional regulation, and post‐transcriptional regulation, thus becoming a new hot spot in cancer research.^6^, ^7^ For example, lncRNA HOTAIR is highly expressed in lung cancer, and its high expression is significantly associated with lung cancer metastasis and poor prognosis 8; lncRNA PVT1 is upregulated in gastric cancer tissues, and its overexpression enhances the cancer cell proliferation and invasion, which is significantly associated with poor prognosis.^9^ Existing studies also report that lncRNA has important functions in HCC. For example, high expression of lncRNA MALAT1, promoting tumor proliferation and metastasis, is expected to be one of the potential HCC therapy targets.^10^ In recent years, the role of lncRNA double homeobox A pseudogene 8 (DUXAP8) has been gradually studied in cancers. Studies show that DUXAP8 exerts a regulatory role in esophageal squamous cell carcinoma, renal cell carcinoma, gastric cancer, and other tumors.^11^, ^12^, ^13^ A recent study reports that in HCC, DUXAP8 repressed tumor suppressor KLF2 transcription through interacting with EZH2.^14^ However, the function and mechanism of DUXAP8 in HCC needs further exploration.

MicroRNAs are widely distributed in eukaryotes and are endogenously expressed noncoding single‐stranded small‐molecule RNAs composed of 19‐25 nucleotide molecules. miRNA regulates the expression of related genes at the post‐transcription or translation, which is at play in the process of tumorigenesis, development, and metastasis.^15^, ^16^, ^17^ Accumulating studies in the past decades have proved that miRNAs can act as tumor suppressors or cancer‐promoting factors in the development of HCC.^18^, ^19^ miR‐422a is abnormally expressed in colorectal cancer, glioma, head and neck squamous cell carcinoma, and counts in the proliferation and metastasis of cancer cells.^20^, ^21^, ^22^ Importantly, miR‐422a is downregulated in HCC, and overexpression can significantly inhibit the malignant phenotypes of HCC cells.^23^ While the mechanism of miR‐422a dysregulation in HCC needs further investigation.

The aim of this work was to investigate the expression and clinical significance of DUXAP8 in HCC, and to explore the effects of DUXAP8 on HCC cells and its downstream mechanisms. Our results proved that DUXAP8 was upregulated in HCC tissues, and patients with high expression of DUXAP8 had poor prognosis, and knocking down DUXAP8 inhibited the malignant phenotypes of HCC cells. We also confirmed that DUXAP8 could promote the development of HCC cells by modulating miR‐422a/pyruvate dehydrogenase kinase 2 (PDK2).

## 2 MATERIALS AND METHODS

### 2.1 Tissue samples

About 50 patients with HCC who received surgery in Jining NO.1 People's Hospital from 2014 to 2018 were selected to obtain cancer tissue samples and adjacent normal tissues. All cases were confirmed by pathological examination. The patients were 31‐73 years old, including 31 males and 19 females. All patients signed informed consents. All specimens were stored in liquid nitrogen at −196°C for RNA extraction. The collection and use of patient tissue samples were approved by the Ethics Committee of Jining NO.1 People's Hospital.

### 2.2 Cell culture

Human normal liver cell line L0‐2, human hepatoma cell lines Huh7, HCCLM3, Hep3B, SMMC‐7721, QSG‐7701 were purchased from Shanghai Institute of Biological Sciences, Chinese Academy of Sciences, and the cells were placed in RPMI‐1640 medium (Gibco) containing 10% fetal bovine serum (FBS; Gibco, Life Technologies) and 1% penicillin/streptomycin (Hyclone) in a constant temperature incubator with 5% CO_2_ at 37°C.

### 2.3 Cell transfection

The cells were washed with PBS buffer, trypsinized for 2 minutes, and transferred to a 15 mL sterile centrifuge tube. Then cells were centrifuged and counted, and inoculated in a 6‐well plate with 1 × 10^5^ cells per well. When the fusion rate was about 70%, transfection was performed. In brief, the transfection reagent (lipofectamine 3000; Thermo Fisher Scientific) was diluted with serum‐free medium, and incubated at 37°C for 20 minutes. The concentration of si‐DUXAP8, DUXAP8 plasmid, miR‐422a mimics, miR‐422a inhibitors, PDK2 siRNA, PDK2 overexpressing plasmid and the control siRNA, plasmids, or miRNA (GenePharma) were also diluted with serum‐free medium, and incubated at room temperature for 5 minutes, and then mixed with the transfection reagent. Then, the cells were incubated with the mixture, and the culture was continued. After 12 hours, the status of the transfected cells was observed, and the serum‐free medium was changed to the complete medium. After the culture was continued for 24 hours, the RNA was extracted and the transfection efficiency was verified by qRT‐PCR.

### 2.4 Quantitative real‐time polymerase chain reaction (qRT‐PCR)

Total RNA was extracted with TRIzol reagent (Invitrogen). Then the total RNA was reversely transcribed into cDNA using First Strand cDNA Synthesis Kit (Thermo Fisher Scientific Inc). PCR was performed using SYBR Green Premix Ex Taq II (TaKaRa) in line with the manufacturer's protocol. The reaction conditions were as follows: pre‐denaturation at 95°C for 30s, denaturation at 95°C for 5 seconds, annealing at 60°C for 30 seconds, 45 cycles. The relative expressions of DUXAP8, miR‐422a, and PDK2 mRNA were obtained by 2^−ΔΔCT^ method. The primers were designed and synthesized by TaKaRa, and the primer sequences are shown in Table 1.

**Table 1:** RT‐qPCR primer sequence

Name	Primer sequences
DUXAP8	Forword:5′‐GATGGTGGAGTAGGAGHCCAG‐3′
	Reverse:5′‐TGHCCCTTCTATTCCACAHCCA‐3′
miR‐422a	Forword:5′‐ACUGGACUUAGGGUCAGAAGHCC‐3′
	Reverse:Uni‐miR qPCR primer
PDK2	Forword:5′‐ATGGCAGTCCTCCTCTCTGA‐3′
	Reverse:5′‐CACCCACCCTCTTCCTAACA‐3′
GAPDH	Forword:5′‐GAAGGTGAAGGTCGGAGTC‐3′
	Reverse:5′‐GAAGATGGTGATGGGATTTC‐3′

### 2.5 Cell counting kit‐8 (CCK‐8) assay

Cell viability was assessed using the CCK‐8 assay. Cells were inoculated at the density of 1 × 10^3^ cells/well in 96‐well plates and cultured for 24, 48, 72, and 96 hours, respectively. According to the manufacturer's instructions, at each time point, 10 μL from CCK‐8 kit (Dojindo Molecular Technologies) was added to each well. After incubation for 1h, the value of OD_450nm_ was measured by a microplate reader (Bio‐Rad).

### 2.6 Scratch healing experiment

The cells in the logarithmic growth phase were plated in a 6‐well plate at a density of 1 × 10^6^/mL. When the cells reached 80% to 90% fusion, a scratch was made with a sterile pipette. Then the cells were gently washed three times with PBS, and the floating cells were removed, and RPMI‐1640 medium containing 2.5% FBS was added. Then under inverted microscope, the scratch width was observed and recorded. After that, the cells were cultured for 24 hours. Ultimately, under inverted microscope, the scratch width was observed and recorded again.

### 2.7 Transwell assay

Cells suspended in RPMI1640 medium containing 10% FBS was added to the wells of 24‐well plate (700 μL/well), and Transwell chambers (8 μm pore size, BD Biosciences) were placed on the wells. The successfully transfected cells were added into the upper compartment of Transwell chamber (2 × 10^5^ cells/chamber). After 24 hours of cell culture, the chambers were removed. The cells failing to pass through the membrane were wiped off with a cotton swab. The migrated or invaded cells were then immersed in 4% paraformaldehyde solution for 10 minutes, and stained with 0.1% crystal violet solution for 30 minutes. After the membrane was washed, cells were observed and counted under a microscope. In invasion assay, Matrigel was used to coat the Transwell chamber before the experiments, and in migration assay, Matrigel was not used.

### 2.8 Dual‐luciferase reporter assay

All luciferase reporter vectors (DUXAP8‐WT, DUXAP8‐MUT) were constructed by Promega. DUXAP8‐WT vector or DUXAP8‐MUT vector, miR‐422a mimics, or negative control miRNAs were co‐transfected into Hep3B cells, and after 48 hours of transfection, the relative luciferase activity was determined using Dual‐luciferase Reporter Assay System (Promega).

### 2.9 Western blot

The cells were lysed with RIPA lysate (containing 1% PMSF), and the supernatant was collected by centrifugation to extract total cellular protein. The protein was quantified by Bradford method. The sample was boiled for 5 minutes, cooled on ice, centrifuged for 30 seconds, and the supernatant was subjected to sodium dodecyl sulfate‐polyacrylamide gel electrophoresis. Then, the protein was transferred to the polyvinylidene fluoride (PVDF) membrane, and then the PVDF membrane was blocked at room temperature with 5% skim milk and incubated overnight at 4°C with primary antibodies. The primary antibodies were anti‐PDK2 (ab68164, Abcam, 1:1000) and anti‐GAPDH (ab8245, Abcam, 1:5000). After the PVDF membrane was washed twice with TBST, the membrane was incubated with fluorescein‐labeled secondary antibody at room temperature for 1 hours. After the membrane was washed with TBST again, color rendering was performed using hypersensitive ECL (Biossci Biotechnology Co, Ltd.).

### 2.10 Statistics analysis

Graphpad Prism (verse 7.0, GraphPad Prism Software) was used for Statistics analysis. All data were showed as mean ± SD. Student's *t* test was used to compare the differences between two groups. The chi‐square test was used to analyze the correlation between the expression of DUXAP8 and the clinical pathological parameters of HCC patients. Survival curves were plotted using the Kaplan‐Meier method and log‐rank tests were performed. Differences of *P < *.05 were considered statistically significant.

## 3 RESULTS

### 3.1 DUXAP8 was upregulated in HCC tissues and cells

**Figure 1:**
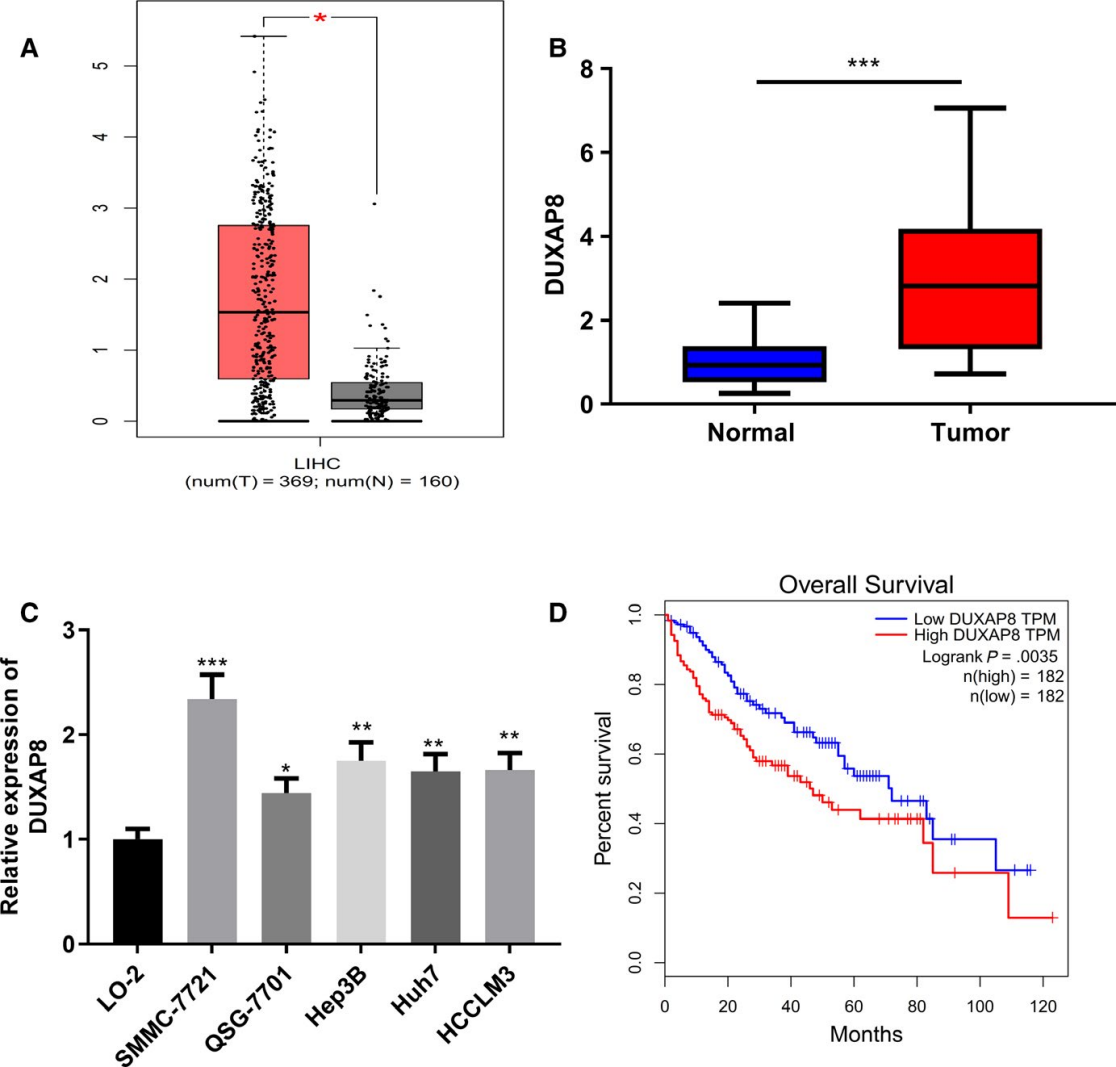
DUXAP8 was upregulated in hepatocellular carcinoma (HCC) tissues and cells. A, Expression levels of DUXAP8 in HCC was analyzed by GEPIA database with TCGA data. B, Expression levels of DUXAP8 in 50 matched HCC tissues and corresponding adjacent liver tissues were detected by qRT‐PCR. C, Expression levels of DUXAP8 in HCC cell lines and normal liver cell line L02 was detected by qRT‐PCR. D, Survival analysis of HCC patients with high and low expression of DUXAP8. *P < .05, **P < .01, ***P < .001

Initially, we searched for changes in the expression of DUXAP8 in HCC samples based on data from GEPIA (http://gepia.cancer-pku.cn/), and it showed that DUXAP8 was upregulated in HCC tissues compared with normal liver tissues (Figure 1A). We also examined the expression of DUXAP8 in 50 pairs of HCC samples and adjacent tissue samples by qRT‐PCR. Consistenly, the results showed that the expression of DUXAP8 in tumor tissues was significantly higher than that in nontumor tissues (Figure 1B). Additionally, we also examined the expression of DUXAP8 in HCC cell lines (SMMC‐7721, QSG‐7701, Hep3B, Huh7, and HCCLM3) and normal human liver cell line L0‐2 by qRT‐PCR. As shown, the expressions of DUXAP8 were significantly increased in HCC cells compared to normal liver cell lines (Figure 1C). To further figure out the relationship between DUXAP8 expression and long‐term prognosis of patients, we performed Kaplan‐Meier analysis based on TCGA patients using GEPIA. As shown, patients with higher DUXAP8 expression levels had shorter overall survival time than patients with lower DUXAP8 expressions (Figure 1D). To further explore the clinical significance of DUXAP8 high expression in HCC, we assessed the correlation between DUXAP8 levels and clinicopathological characteristics of patients. To this end, 50 patients were divided into two groups with high or low DUXAP8 expressions. Chi‐squared test showed that high expression of DUXAP8 was significantly associated with larger tumor size (*P* = .0227), TNM stage (*P* = .0072), and distant metastasis (*P* = .0018). However, there was no significant relationship between DUXAP8 expression and other factors including gender and age (Table 2). These results suggested that DUXAP8 was involved in the progression of HCC.

**Table 2:** Correlation between clinicopathological characteristics and lncRNA DUXAP8 expression levels in hepatocellular carcinoma (HCC) patients

Characteristics	Number of patients	DUXAP8 expression		*χ^2^*	*P* value
		Low	High		
Gender					
Male	31	18	13	2.1222	.1452
Female	19	7	12		
Age(y)					
<55	29	15	14	0.0821	.7744
≥55	21	10	11		
Tumor size (cm)					
<5	28	10	18	5.1948	.0227
≥5	22	15	7		
AEP (ng/mL)					
<20	19	11	8	0.7640	.3821
≥20	31	14	17		
HBV infection					
Yes	24	14	10	1.2821	.2575
No	26	11	15		
Histological grade					
Well/ Moderate	21	12	9	0.7389	.3900
Poor	29	13	16		
TNM stage					
I + II	17	13	4	7.2193	.0072
III + IV	33	12	21		
Metastasis					
Yes	27	8	19	9.7424	.0018
No	23	17	6		

### 3.2 DUXAP8 promoted the proliferation, migration, and invasion of HCC cells

**Figure 2:**
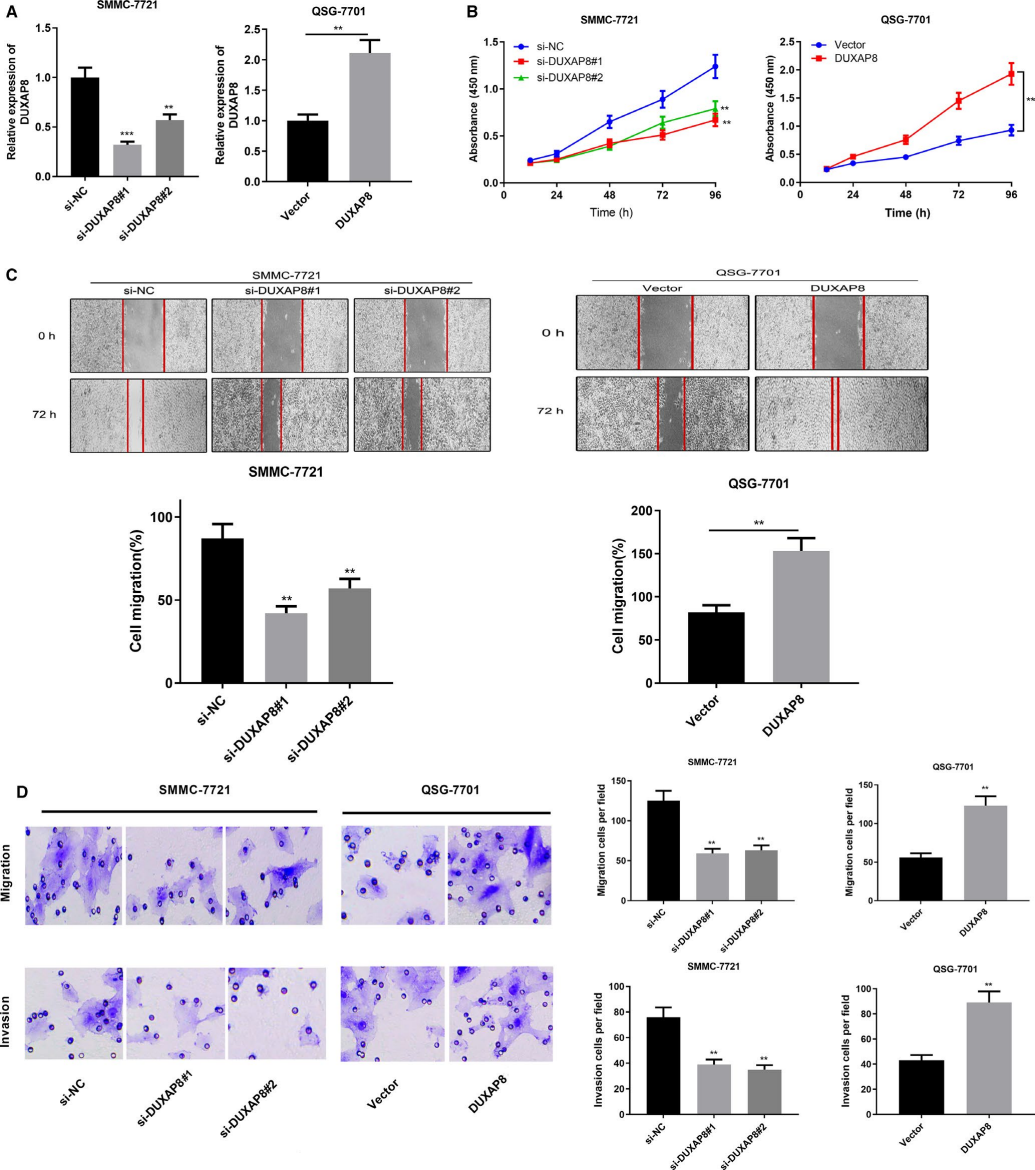
DUXAP8 promoted hepatocellular carcinoma (HCC) proliferation, migration, and invasion. A, Cell lines with low or high expression of DUXAP8 were successfully established. B, The proliferation of HCC cells after DUXAP8 knockdown or overexpression was detected by CCK‐8 assay. C, The migration of HCC cells after DUXAP8 knockdown or overexpression was detected by scratch healing assay. D, The migration and invasion of HCC cells after DUXAP8 knockdown or overexpression was detected by Transwell assay. *P < .05, **P < .01, ***P < .001

**Figure 3:**
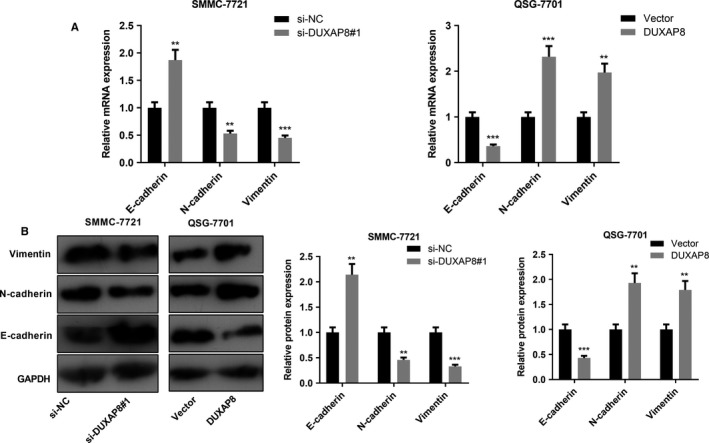
DUXAP8 regulated EMT of hepatocellular carcinoma (HCC) cells. A, The EMT‐related markers E‐cadherin, N‐cadherin, and Vimentin in HCC cells after DUXAP8 knockdown or overexpression were detected by qRT‐PCR. B, The expression levels of E‐cadherin, N‐cadherin, and Vimentin in HCC cells after DUXAP8 knockdown or overexpression were detected by Western blot. **P < .01, ***P < .001

To investigate the biological function of DUXAP8 in HCC cells, we knocked down or overexpressed DUXAP8 in SMMC‐7721 and QSG‐7701 cells by transfection with siRNA or overexpressing plasmids (Figure 2A). Proliferation was detected by CCK‐8 assay. The results showed that DUXAP8 knockdown significantly inhibited SMMC‐7721 cell proliferation, while DUXAP8 overexpression promoted QSG‐7701 cell proliferation (Figure 2B). We further examined the migration ability of SMMC‐7721 and QSG‐7701 in HCC cells by scratch assay. The results showed that the migration ability of SMMC‐7721 cells was significantly decreased after knockdown of DUXAP8, while overexpression of DUXAP8 significantly enhanced the migration ability of QSG‐7701 cells (Figure 2C). We also examined the effects of DUXAP8 on migration and invasion of HCC cells by Transwell assay. Consistently, the results demonstrated that knockdown of DUXAP8 inhibited the migration and invasion of SMMC‐7721 cells; in contrast, overexpression of DUXAP8 promoted the migration and invasion of QSG‐7701 cells (Figure 2D). We then detected the expression of epithelial‐mesenchymal transition (EMT)‐related markers E‐cadherin, N‐cadherin, and Vimentin in SMMC‐7721 and QSG‐7701 lines with qRT‐PCR and Western blot. As expected, after DUXAP8 knockdown, N‐cadherin and Vimentin were downregulated while E‐cadherin was upregulated, whereas overexpression of DUXAP8 displayed the opposite effect (Figure 3A). Collectively, these results confirmed that DUXAP8 was an oncogenic lncRNA in HCC.

### 3.3 DUXAP8 targeted miR‐422a in HCC cells

**Figure 4:**
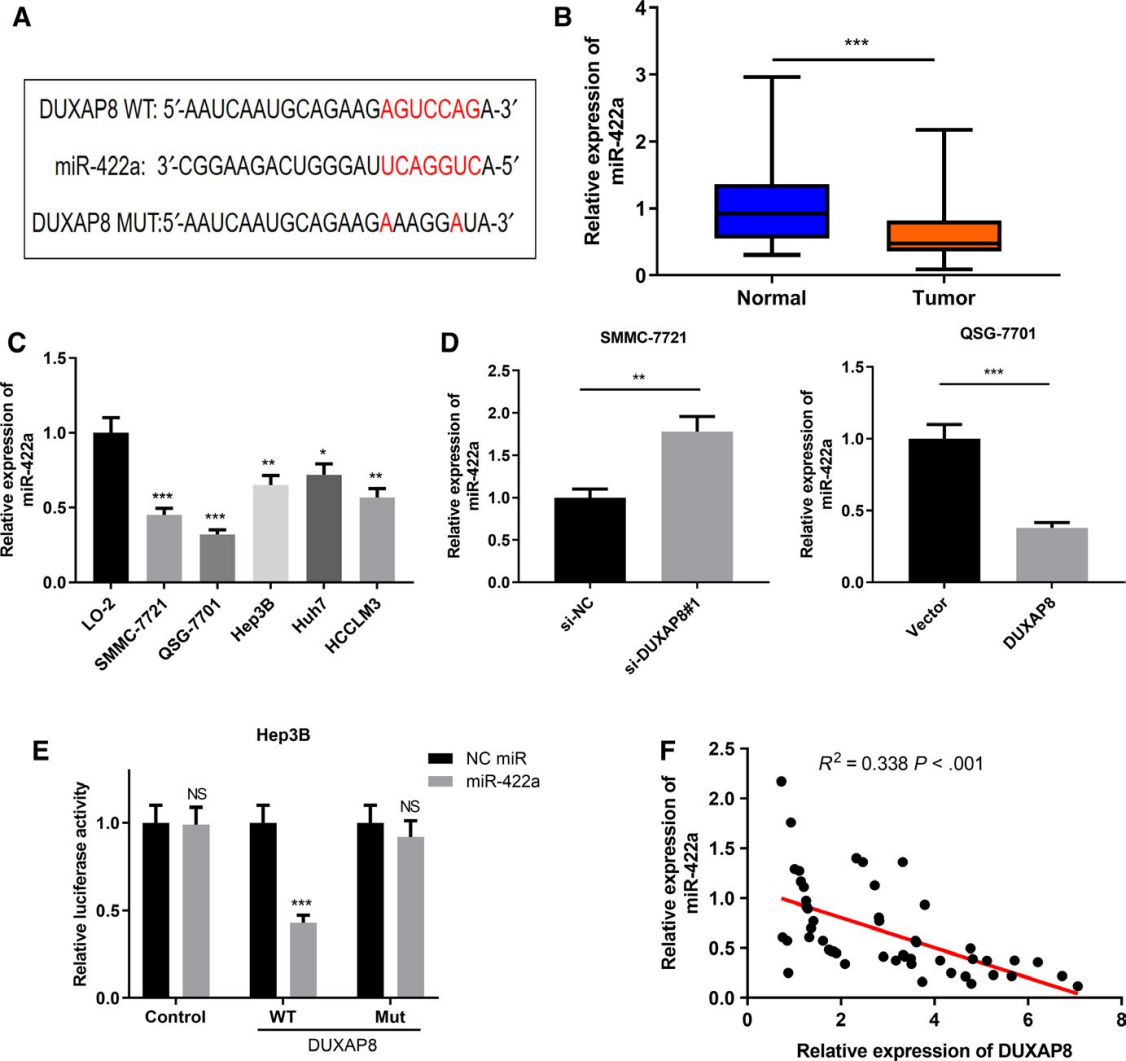
DUXAP8 targeted miR‐422a in hepatocellular carcinoma (HCC). A, Prediction of binding sites between miR‐422a and DUXAP8. B, The expression levels of miR‐422a in 50 matched HCCs and adjacent nontumor controls were detected by qRT‐PCR. C, The expression of miR‐422a in HCC cell lines and normal liver cell line L02 was detected by qRT‐PCR. D, The expression levels of miR‐422a in HCC cell lines were detected by qRT‐PCR after DUXAP8 knockdown or overexpression. E, Dual‐luciferase reporter assay indicated that miR‐422a mimics could reduce the luciferase activity of wild‐type DUXAP8 reporter. F, The correlation between the expression levels of DUXAP8 and miR‐422a in HCC samples was analyzed. *P < .05, **P < .01, ***P < .001, NS: *P* > .05

LncRNA can act as a molecular sponge to regulate the expression of downstream genes through absorbing miRNA. By searching for the potential binding miRNA of DUXAP8 in the StarBase online database (http://www.starbase.sysu.edu.cn), miR‐422a was selected as a predictive target for DUXAP8 because of its high binding potential (Figure 4A). Furthermore, the expression of miR‐422a was significantly downregulated in HCC tissues and cells (Figure 4B,C). Additionally, an increase in miR‐422a expression was observed in SMMC‐7721 cells with DUXAP8 knockdown, whereas the expression of miR‐422a was decreased in QSG‐7701 cells with DUXAP8 overexpression, suggesting that DUXAP8 can negatively regulate miR‐422a expressions in HCC (Figure 4D). To further validate the binding relationship between miR‐422a and DUXAP8, dual‐luciferase reporter assay was performed and it showed that luciferase activity decreased significantly after the Hep3B cells were co‐transfected with miR‐422a and DUXAP8‐WT reporter plasmids; however, the luciferase activity did not decrease significantly when the cells were co‐transfected with miR‐422a and DUXAP8‐MUT reporter plasmids (Figure 4E). Importantly, the expression correlation between DUXAP8 and miR‐422a was analyzed in HCC tissue samples and their expression was found to be negatively correlated (Figure 4F). Taken together, these results indicated that DUXAP8 negatively regulated miR‐422a in HCC.

### 3.4 miR‐422a could reverse the function of DUXAP8 on HCC cells

**Figure 5:**
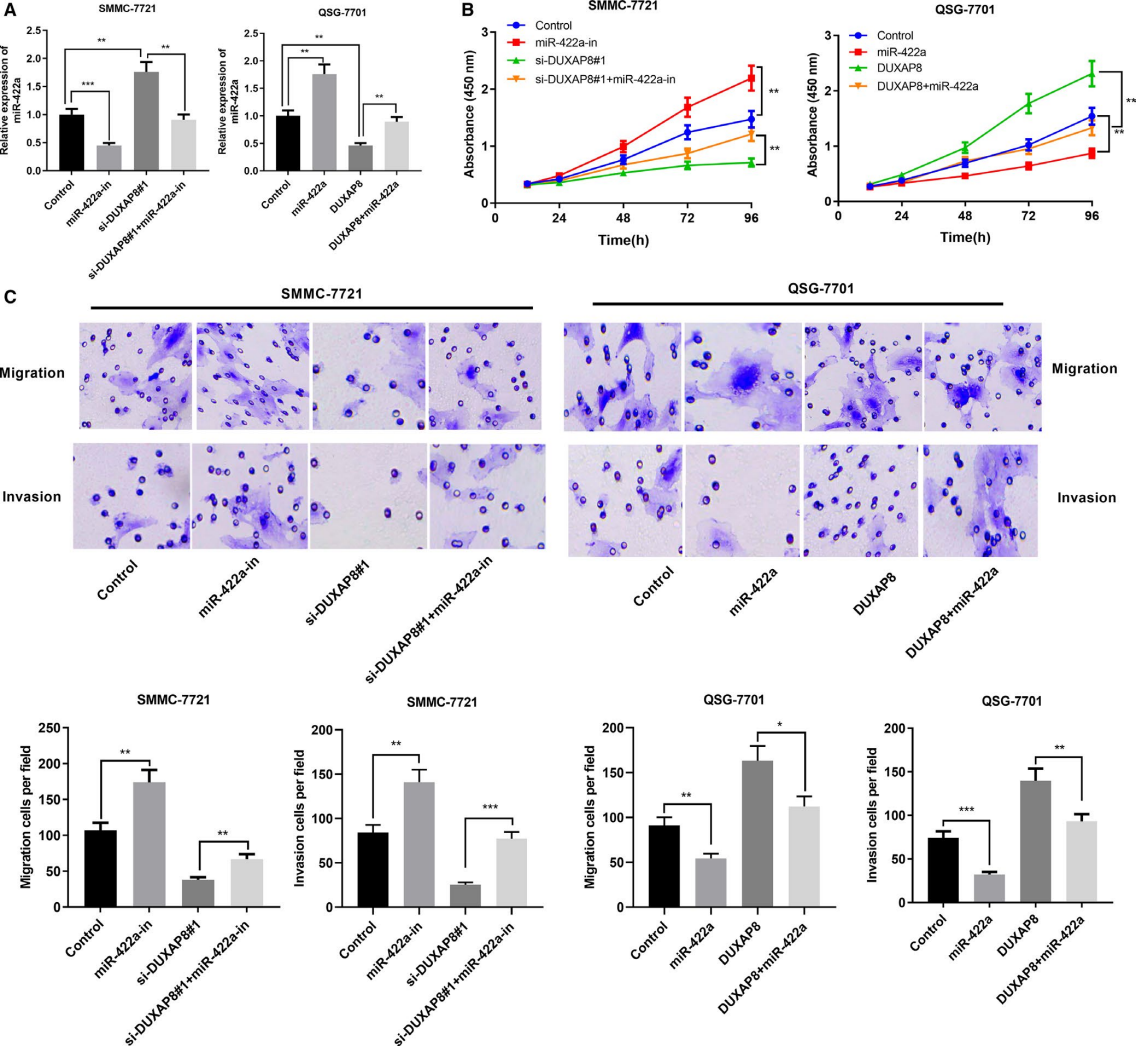
miR‐422a could reverse the function of DUXAP8 in hepatocellular carcinoma (HCC). A, miR‐422a mimics or inhibitors and DUXAP8 siRNA were co‐transfected into HCC cells, and the expression of miR‐422a was detected by qRT‐PCR. B, The proliferation of HCC cells in each group was detected by CCK‐8 assay. C, The migration and invasion of HCC cells in each group were evaluated by Transwell assay. *P < .05, **P < .01, ***P < .001

Next, we transfected the miR‐422a inhibitors or mimics into SMMC‐7721 and QSG‐7701 of HCC cells with DUXAP8 knockdown or overexpression. We found that transfection of miR‐422a inhibitors enhanced the upregulation of miR‐422a expression caused by DUXAP8 knockdown. In contrast, transfection of miR‐422a mimics attenuated downregulation of miR‐422a expression by DUXAP8 overexpression (Figure 5A). Then CCK‐8 and Transwell assays were conducted. As shown, the inhibition of miR‐422a reversed the effect of DUXAP8 knockdown on the proliferation, migration, and invasion of HCC cells; overexpression of miR‐422a attenuated the effect of overexpressing DUXAP8 on proliferation, migration, and invasion of HCC cells (Figure 5B,C). These findings indicated that miR‐422a reversed the function of DUXAP8 in HCC cells.

### 3.5 Regulation of DUXAP8/miR‐422a on PDK2 expression

**Figure 6:**
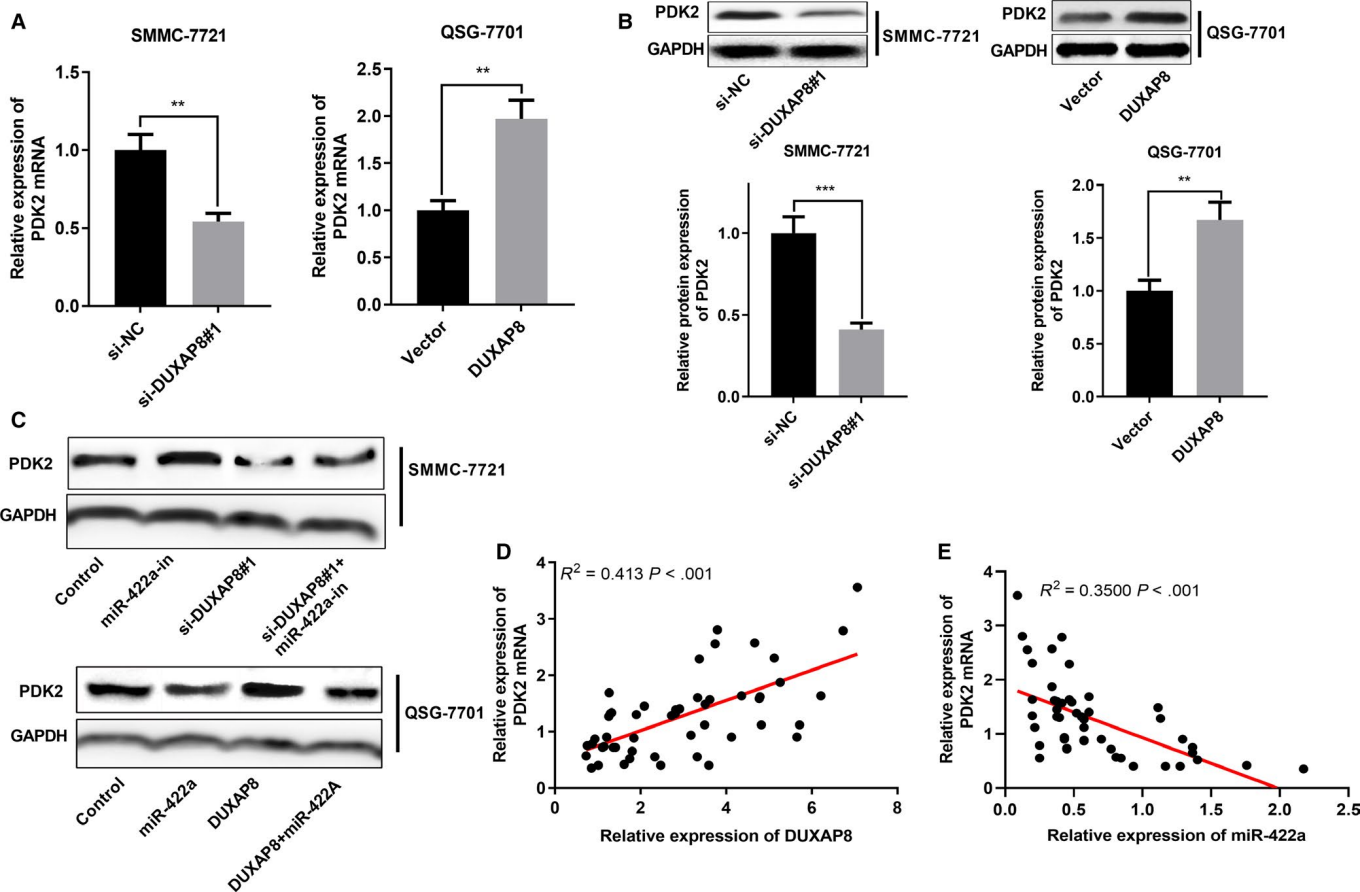
Regulation of DUXAP8/miR‐422a on PDK2 expressions. A, qRT‐PCR was used to detect the effect of knockdown or overexpression of DUXAP8 on PDK2 mRNA expressions in hepatocellular carcinoma (HCC) cell lines. B, Western blot was used to detect the effect of knockdown or overexpression of DUXAP8 on PDK2 protein expressions in HCC cell lines. C, Western blot was used to detect the effect of DUXAP8 and miR‐422a on PDK2 protein expressions. D, The correlation between the expression levels of DUXAP8 and PDK2 mRNA in HCC samples was analyzed. E, The correlation between the expression levels of miR‐422a and PDK2 mRNA in HCC samples was analyzed. **P < .01, ***P < .001

After confirming that DUXAP8 can regulate miR‐422a expression, we attempted to explore the downstream targets of miR‐422a in HCC. A recent study demonstrates that PDK2, an oncogene, is a target of miR‐422a,^24^ so we investigated whether DUXAP8 can regulate PDK2 expression in HCC. As shown, DUXAP8 knockdown significantly reduced mRNA and protein expressions of PDK2, and overexpression of DUXAP8 increased mRNA and protein expressions of PDK2 (Figure 6A,B). Furthermore, our results revealed that inhibition of miR‐422a enhanced the PDK2 expression and reversed the effect of DUXAP knockdown on PDK2. Conversely, overexpression of miR‐422a reduced the PDK2 expression and abolished the DUXAP8‐induced upregulation of PDK2 (Figure 6C). Furthermore, we also found that the expression of PDK2 mRNA and DUXAP8 were positively correlated in HCC tissue samples, while the expression of PDK2 mRNA was negatively correlated with the expression of miR‐422a (Figure 6D,E). Additionally, Western blot also indicated that both miR‐422a and PDK2 could modulate the process of EMT of HCC cells (Figures S1 and Figure S2). These results indicated that DUXAP8 could upregulate PDK2 expression in HCC and promote HCC progression, probably by modulating miR‐422a.

## 4 DISCUSSION

The exact molecular mechanism of HCC is still unclear.^1^ The pathogenesis is a complex process with multiple factors and steps. Growing studies have shown that lncRNAs are abnormally expressed in human tumors, including HCC.^10^, ^11^ Therefore, the identification of key lncRNAs associated with HCC is critical to clarify this disease and identify novel therapeutic targets. It is reported that lncRNA GAS5 is downregulated in HCC, and its overexpression can impede cell migration and invasion 25; the expression of lncRNA HOTAIR in HCC is elevated, and patients with high expression of it have a poor prognosis 26; moreover, the expression of lncRNA MALAT1 is also confirmed to be an independent prognostic factor for HCC.^10^ It is worth noting that the expression of DUXAP8 is closely related to the occurrence and progression of a variety of tumors. For example, DUXAP8 is upregulated in esophageal squamous cell carcinoma, and its expression is positively correlated with TNM stage and lymph node metastasis, and negatively correlated with patients’ survival rate 12; DUXAP8 is highly expressed in bladder cancer tissues, and knocking down its expression can reduce the cell viability.^27^ In this study, we demonstrated that DUXAP8 was upregulated in HCC tissues and cells, and its high expression was associated with several adverse clinical features, including increased tumor size, distant metastasis, and increased TNM staging. We also proved that overexpression of DUXAP8 significantly promoted the proliferation, metastasis, and EMT of HCC cells; DUXAP8 knockdown repressed the malignant phenotypes of HCC cells. These data were consistent with previous report,^14^ confirming DUXAP8 as an oncogenic lncRNA in HCC.

miRNAs are at play in the progression of a variety of tumors.^28^ Studies have found that miRNAs also mattered in the occurrence and development of HCC. For example, miR‐27b is significantly upregulated in HCC tissues and cells, and its high expression is significantly associated with poor tumor differentiation and vascular invasion; inhibition of miR‐27b can inhibit migration and invasion of HCC cells.^29^ The expression of miR‐138 was significantly downregulated in HCC tissues and cells, and its overexpression significantly impeded cell proliferation.^30^ Increasing studies have shown that lncRNA can repress miRNAs as competitive endogenous RNAs (ceRNAs). It is reported that DUXAP8 can act as a ceRNA of miR‐577 to promote the expression of RAB14, thus promoting the migration and invasion of colorectal cancer cells 31; in renal cell carcinoma, DUXAP8 targets miR‐126 and promotes cancer progression.^13^ Based on the difference in the expression and role of DUXAP8 and miR‐422a in HCC, we speculated that there was a regulatory relationship between them. We validated the binding relationship between DUXAP8 and miR‐422a by dual‐luciferase reporter gene assay. Additionally, overexpression of DUXAP8 significantly reduced the expression of miR‐422a in HCC, while its knockdown induced the expression of miR‐422a. Importantly, with functional experiments, we demonstrated that miR‐422a could reverse the function of DUXAP8 on HCC cells. Our results not only explained the mechanism of miR‐422a dysregulation in HCC, but also proved that miR‐422a was a crucial effector during DUXAP8 regulating the malignant phenotypes of HCC cells.

Metabolic reprogramming is a common feature of cancer cells, and a shift from mitochondrial oxidative phosphorylation to aerobic glycolysis is involved in the tumorigenesis and cancer progression (well known as “Warburg effect”).^32^ PDK2 belongs to the pyruvate dehydrogenase kinase family. It can downregulate the activity of the mitochondrial pyruvate dehydrogenase complex.^32^, ^33^ By reducing the conversion of pyruvate to acetyl‐CoA, PDK2 switches carbon flow from the tricarboxylic acid cycle and de novo lipogenesis to lactate production, thus facilitating Warburg effect.^33^ Dysregulation of PDK2 is reported in multiple cancers, and linked to proliferation, metastasis, and chemoresistance of cancer cells.^34^, ^35^, ^36^, ^37^ Specifically, in HCC, PDK2 is identified as target of miR‐214, and involved in disease progression.^37^ Recently, PDK2 has been proved to be negatively regulated by miR‐422a, and PDK2 inhibition by miR‐422a results in increased activity of the pyruvate dehydrogenase and higher acetyl‐CoA level.^24^ We found that PDK2 can be enrolled in the development of HCC cells as a carcinogenic factor and may be one of the candidate targets for miR‐422a. Overexpression of DUXAP8 significantly promoted PDK2 mRNA and protein expression, while knockdown of DUXAP8 had the opposite effect, and DUXAP8 was positively correlated with PDK2 expression in HCC tissues. Interestingly, expression of miR‐422a partially reversed the promotion of PDK2 expression caused by DUXAP8. These results manifested that DUXAP8 can target miR‐422a to indirectly regulate PDK2 and thus affect the development of HCC cells.

This study has several shortcomings. In the first place, in vivo experiments are essential to further verify our results. In addition, other downstream miRNAs of DUXAP8 needs to be screened and validated, which can better clarify the mechanism by which DUXAP8 promotes cancer progression. What's more, as mentioned above, upregulation of PDK2 is associated with metabolic reprogramming and chemoresistance of cancer cells, so it is also worth studying whether DUXAP8 can modulate these phenotypes of HCC cells.

In conclusion, DUXAP8 is upregulated in HCC tissues and cells. Overexpression of DUXAP8 significantly promotes proliferation, metastasis, and EMT of HCC cells, while knockdown inhibits the malignant phenotype of HCC cells. This study also elucidates the mechanism of DUXAP8/miR‐422a/PDK2 axis in the development of HCC. With the deepening of research, DUXAP8 is likely to become a marker for clinical diagnosis and prognosis, and even be a therapeutic target of HCC.

## CONFLICT OF INTEREST

The authors declare that there are no conflict of interest.

## Data Availability

The data used to support the findings of this study are available from the corresponding author upon a reasonable request.
