# Supplementary figures and images for: Long noncoding RNA DUXAP8 contributes to the progression of hepatocellular carcinoma via regulating miR‐422a/PDK2 axis
Source: Cancer Med. 2020 Feb 5;9(7):2480–90. doi: 10.1002/cam4.2861 (PMC7131864; doi:10.1002/cam4.2861)

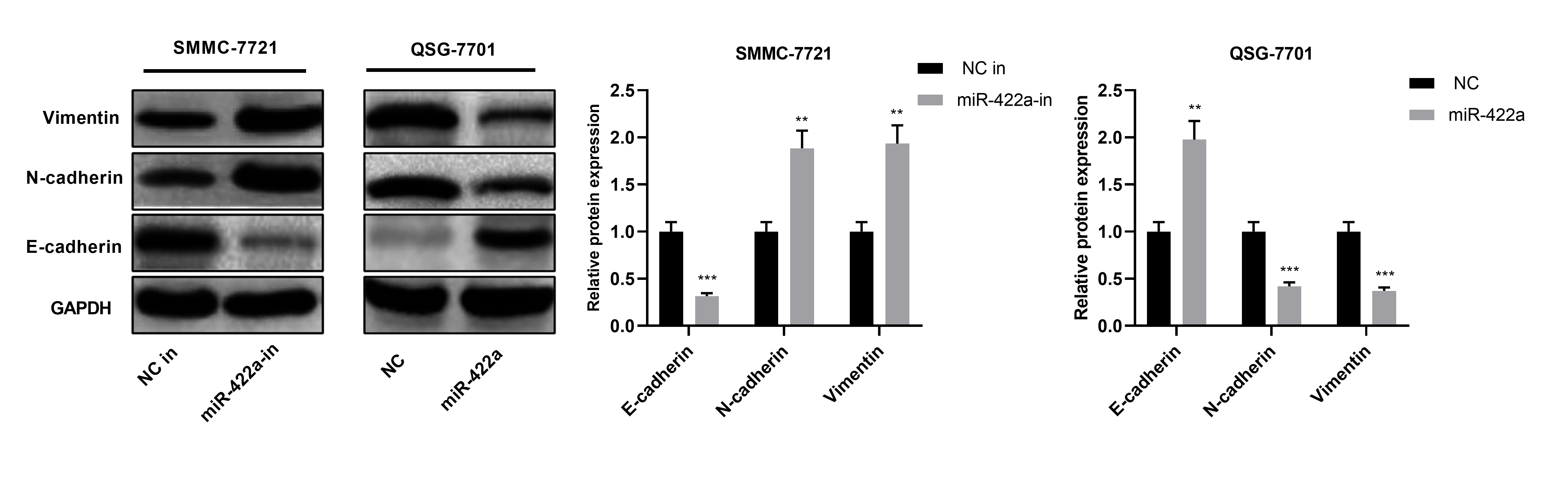

Supplement: Supplementary file 1 [file CAM4-9-2480-s001.tif]

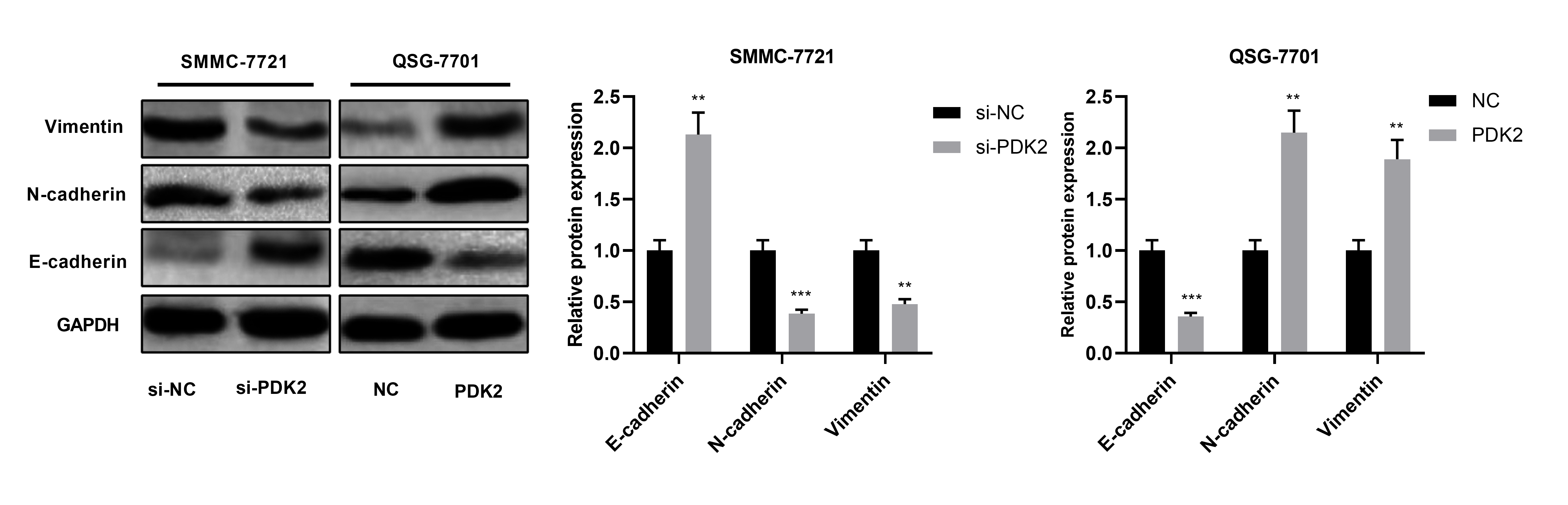

Supplement: Supplementary file 2 [file CAM4-9-2480-s002.tif]
